# Supplementary material for: Identifying Essential Hub Genes and circRNA-Regulated ceRNA Networks in Hepatocellular Carcinoma
Source: Int J Mol Sci. 2025 Feb 7;26(4):1408. doi: 10.3390/ijms26041408 (PMC11855757; doi:10.3390/ijms26041408)
Supplement: Supplementary file 1 [file ijms-26-01408-s001.zip › ijms-3437435-supplementary/Supplementary Figure 1=2025-02-05.pdf]

Supplementary Figure 1

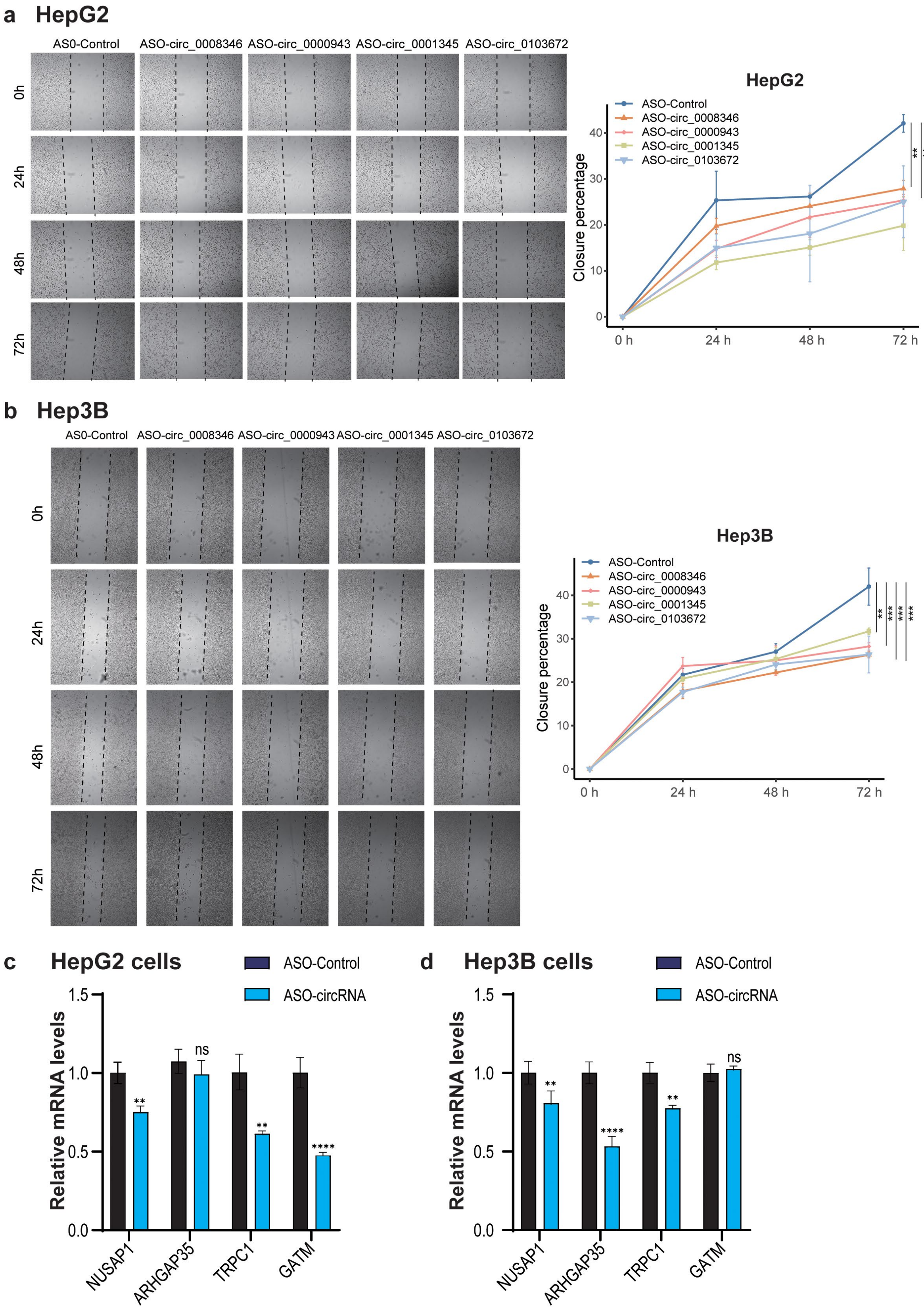

Supplementary Figure 1. Scratch assays and effects of circRNA knockdown on the expression of corresponding cognate protein-coding genes. a, **b** Scratch assays to evaluate cell migrating ability with knockdown of 4 selected circRNAs in HepG2 (a) and Hep3B cells (b). The quantified percentages of closure from three independent

experiments are shown at right. c RT-qPCR analysis of the effects of circRNA knockdown by ASOs on corresponding protein coding genes. The circ RNAs circ_0008346, circ_0000943, circ_0001345 and circ_0103672 can be generated by the mRNAs of protein-coding genes NUSAP1, ARHGAP35, TRPC1 and GATM, respectively. ns, no significance, $P > 0.05$; ** $P < 0.01$; **** $P < 0.001$.
